# Supplementary material for: Comparative characterization of the infant gut microbiome and their maternal lineage by a multi-omics approach
Source: Nat Commun. 2024 Apr 8;15:3004. doi: 10.1038/s41467-024-47182-y (PMC11001937; doi:10.1038/s41467-024-47182-y)
Supplement: Supplementary file 1 — Supplementary Information [file 41467_2024_47182_MOESM1_ESM.pdf]

Supplementary Information

Comparative characterization of the infant gut microbiome and their maternal lineage by a multi-omics approach.

Authors: Tomás Clive Barker-Tejeda[1,2†]; Elisa Zubeldia-Varela[1,2†]; Andrea Macías-Camero[1,2†]; Lola Alonso[3]; Isabel Adoración Martín-Antoniano[2,3,4]; María Fernanda Rey-Stolle[1]; Leticia Mera-Berriatua[2]; Raphaëlle Bazire[5,6]; Paula Cabrera-Freitag[7]; Meera Shanmuganathan[8]; Philip Britz-McKibbin[8]; Carles Ubeda[9,10]; M. Pilar Francino[10,11]; Domingo Barber[2]; María Dolores Ibáñez-Sandín[5,6]; Coral Barbas[1]; Marina Pérez-Gordo[2*]; Alma Villaseñor[1,2*]

Affiliations:

[1]Centro de Metabolómica y Bioanálisis (CEMBIO), Facultad de Farmacia, Universidad San Pablo-CEU, CEU Universities, Boadilla del Monte, Spain.

[2]Departamento de Ciencias Médicas Básicas, Instituto de Medicina Molecular Aplicada (IMMA) Nemesio Díez, Facultad de Medicina, Universidad San Pablo-CEU, CEU Universities, Boadilla del Monte, Spain.

[3]Genetic and Molecular Epidemiology Group, Spanish National Cancer Research Centre (CNIO), Madrid, Spain.

[4]Instituto de Estudios de las Adicciones IEA-CEU, Universidad San Pablo-CEU, CEU Universities, Madrid, Spain

[5]Department of Allergy, Hospital Infantil Niño Jesús, Fib-HNJ, Madrid, Spain.

[6]Instituto de Investigación Sanitaria-La Princesa, Madrid, Spain.

[7]Pedriatic Allergy Unit, Allergy Service, Hospital General Universitario Gregorio Marañón, and Gregorio Marañón Health Research Institute, Madrid, Spain.

[8]Department of Chemistry and Chemical Biology, McMaster University, Hamilton, ON, Canada.

[9]Fundació per al Foment de la Investigació Sanitària i Biomèdica de la Comunitat Valenciana (FISABIO), Valencia, Spain

[10]CIBER en Epidemiología y Salud Pública, Madrid, Spain

[11]Joint Research Unit in Genomics and Health, Fundació per al Foment de la Investigació Sanitària i Biomèdica de la Comunitat Valenciana (FISABIO) and Institut de Biologia Integrativa de Sistemes (Universitat de València / Consejo Superior de Investigaciones Científicas), València, Spain

[†]: These Authors have equally contributed

[*]: Corresponding authors

Corresponding authors:

[*]Alma Villaseñor, PhD

CEMBIO, Centro de Metabolómica y Bioanálisis, Facultad de Farmacia, Universidad San Pablo CEU. Avda. Montepríncipe s/n, 28668 Boadilla del Monte, Madrid, SPAIN.

E-mail: alma.villasenor@ceu.es

[*]Marina Pérez-Gordo, PhD

Instituto de Medicina Molecular Aplicada (IMMA) Nemesio Díez, Departamento de Ciencias Médicas Básicas, Facultad de Medicina, Universidad San Pablo CEU, 28660 Madrid, Spain

E-mail: marina.perezgordo@ceu.es

Supplementary Figure Titles:

Figure S1: Justification of sample numbers and outliers in the different steps.

Figure S2: Graphical representation of age distributions and age outliers in all techniques.

Figure S3: Quality Assurance and normalizations in GC-QTOF-MS analysis.

Figure S4: MSI-CE-MS workflow and multivariate models.

Figure S5: Correlations between the metabolites that were measured in both techniques (MSI-CE-MS and GC-QTOF-MS).

Figure S6: Supplementary statistics figures for metabolomics.

Figure S7: Taxonomic composition of faecal samples using 16S rRNA and shotgun gene sequencing.

Figure S8: Correlations between metabolites and bacterial taxa that are known producers of these metabolites.

Figure S9: Cytoscape representation of the links between ASVs, KOs, and metabolites obtained from mixOmics and DIABLO.

Figure S10: Experimental design of the QCs preparation and worklists for GC-QTOF-MS equipment used for large-scale sample analysis.

Supplementary Tables:

Table S1: Main characteristics for each age group, with their descriptive statistics.

Table S2: Pathway analysis of the 88 significant metabolites associated with age after application of the linear mixed effect model with the correction for multiple test comparisons FDR p value < 0.05. Significant and relevant metabolic pathways for the host-microbiota crosstalk are shown according to their FDR p-value ≤ 0.05.

Table S3: Correlations between gamma-aminobutyric acid (GABA) and bacterial phyla, genera, and species using spearman's rank correlation coefficient.

Table S4: Correlations between short-chain fatty acids (SCFA) and bacterial genera and species using spearman's rank correlation coefficient.

Table S5: Correlations between the matched bacterial genera between 16S rRNA gene sequencing and shotgun sequencing using two-sided Pearson correlation test.

Table S6: Description of the KOs from Heatmap and circosplot from Figure 9.

Supplementary Data files:

Supplementary Data 1: Significant identified metabolites detected in the GC-QTOF-MS analysis, with their chemical information, quality parameters and statistics. The statistical test applied was linear mixed-effects model with the correction for multiple test comparisons, FDR p-value < 0.05.

Supplementary Data 2: Significant metabolites detected in the MSI-CE-TOF-MS analysis, with their chemical information, quality parameters and statistics. The statistical test applied was linear mixed-effects model with the correction for multiple test comparisons, FDR p-value < 0.05.

Supplementary Data 3: Comparisons between the bacterial phyla and genera detected by 16S rRNA gene and shotgun sequencing. Data used for the stacked bars in Figure 7A-D.

Supplementary Data 4: Significant results at phylum level for 16S rRNA gene and shotgun sequencing. The statistical test applied was linear mixed-effects model with the correction for multiple test comparisons, FDR p-value < 0.05.

Supplementary Data 5: Significant results at genus level for 16S rRNA gene and shotgun sequencing. The statistical test applied was linear mixed-effects model with the correction for multiple test comparisons, FDR p-value < 0.05.

Supplementary Data 6: Significant results at species level for shotgun sequencing. The statistical test applied was linear mixed-effects model with the correction for multiple test comparisons, FDR p-value < 0.05.

Supplementary Data 7: Significant results from the linear mixed effects model for the KEGG Orthologs (KOs).

Supplementary Data 8: Significant results from the linear mixed effects model for the pathways at BRITE level B.

Supplementary Data 9: Significant results from the linear mixed effects model for the pathways at BRITE level C.

Supplementary Data 10: Detailed information for each sample, including epidemiological variables, techniques in which it was measured and models where it is included.

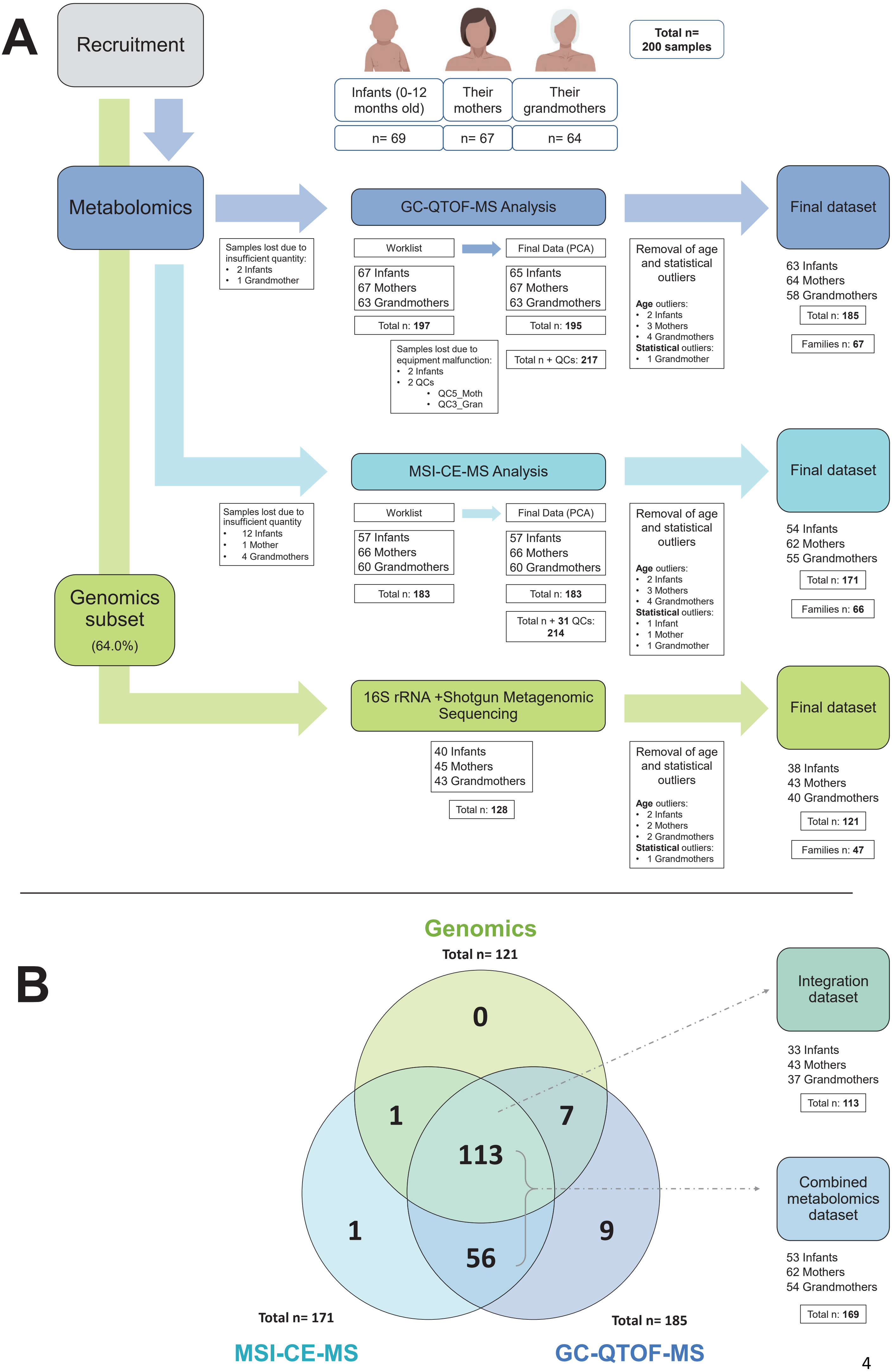
A
Recruitment
Infants (0-12 months old)
Their mothers
Their grandmothers
n= 69
n= 67
n= 64
Total n= 200 samples
Metabolomics
Samples lost due to insufficient quantity: • 2 Infants • 1 Grandmother
GC-QTOF-MS Analysis
Worklist
67 Infants
67 Mothers
63 Grandmothers
Total n: 197
Final Data (PCA)
65 Infants
67 Mothers
63 Grandmothers
Total n: 195
Total n + QCs: 217
Samples lost due to equipment malfunction: • 2 Infants • 2 QCs • QC5_Moth • QC3_Gran
Removal of age and statistical outliers
Age outliers: • 2 Infants • 3 Mothers • 4 Grandmothers
Statistical outliers: • 1 Grandmother
Final dataset
63 Infants
64 Mothers
58 Grandmothers
Total n: 185
Families n: 67
MSI-CE-MS Analysis
Samples lost due to insufficient quantity • 12 Infants • 1 Mother • 4 Grandmothers
Worklist
57 Infants
66 Mothers
60 Grandmothers
Total n: 183
Final Data (PCA)
57 Infants
66 Mothers
60 Grandmothers
Total n: 183
Total n + 31 QCs: 214
Removal of age and statistical outliers
Age outliers: • 2 Infants • 3 Mothers • 4 Grandmothers
Statistical outliers: • 1 Infant • 1 Mother • 1 Grandmother
Final dataset
54 Infants
62 Mothers
55 Grandmothers
Total n: 171
Families n: 66
Genomics subset
(64.0%)
16S rRNA +Shotgun Metagenomic Sequencing
40 Infants
45 Mothers
43 Grandmothers
Total n: 128
Removal of age and statistical outliers
Age outliers: • 2 Infants • 2 Mothers • 2 Grandmothers
Statistical outliers: • 1 Grandmothers
Final dataset
38 Infants
43 Mothers
40 Grandmothers
Total n: 121
Families n: 47
B
Genomics
Total n= 121
0
1
7
113
1
9
56
Total n= 171
Total n= 185
MSI-CE-MS
GC-QTOF-MS
Integration dataset
33 Infants
43 Mothers
37 Grandmothers
Total n: 113
Combined metabolomics dataset
53 Infants
62 Mothers
54 Grandmothers
Total n: 169

Figure S1: Justification of sample numbers and outliers in the different steps. A: Workflow of the study, from recruitment to the final dataset of each experiment. The number of samples and the explanation for the lost samples is given in each step. Age outliers are further detailed in Figure S2. **B:** Venn diagram of the samples measured in all techniques. The 113 common samples were the ones selected for mixOmics integration, and the 169 common in both metabolomics platforms were used for the combined metabolomics dataset. Source data are provided as a Source Data file. Icons were created using biorender.com.

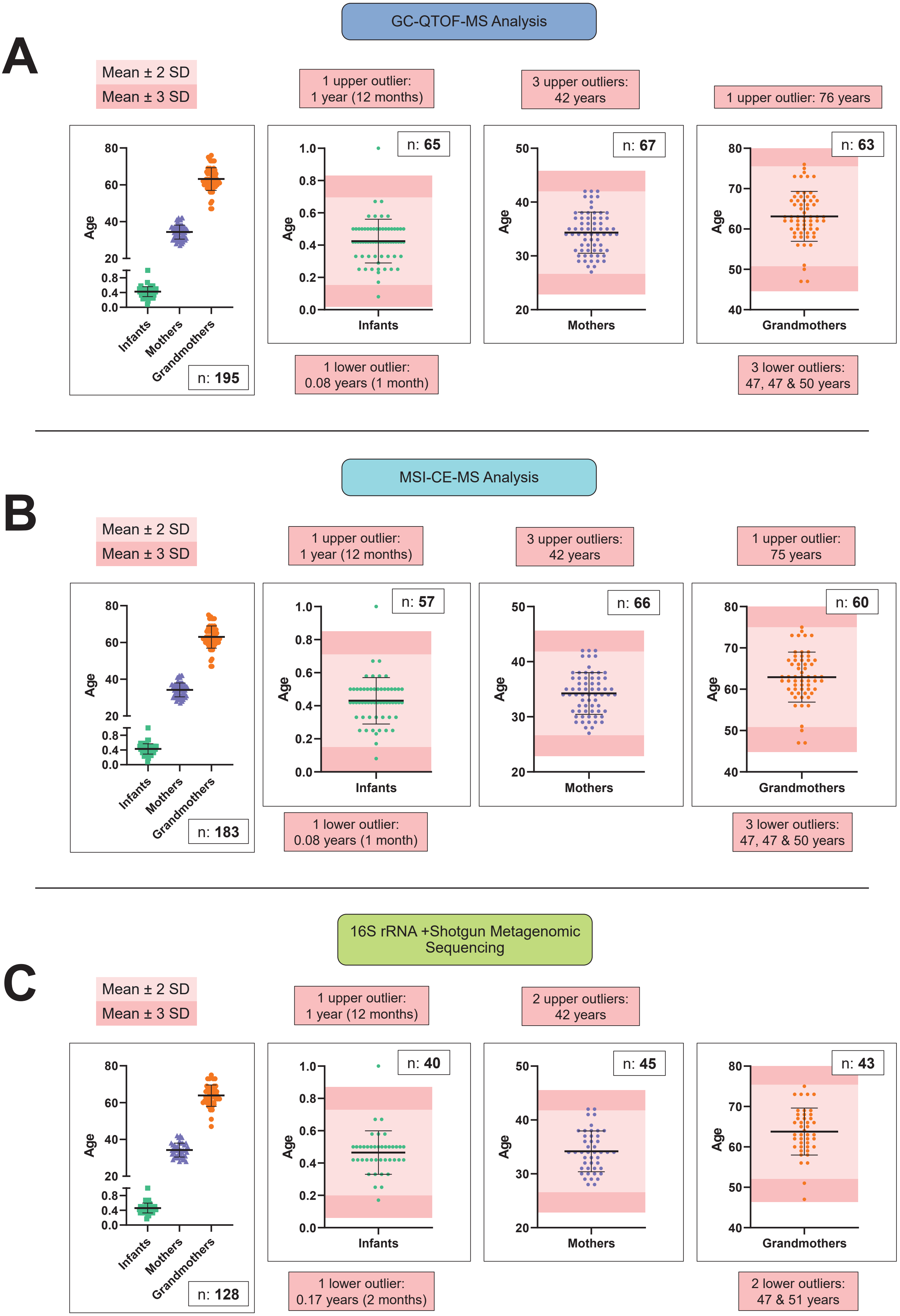
A
GC-QTOF-MS Analysis
Mean ± 2 SD
Mean ± 3 SD
1 upper outlier: 1 year (12 months)
3 upper outliers: 42 years
1 upper outlier: 76 years
n: 195
n: 65
n: 67
n: 63
Age
Infants
Mothers
Grandmothers
1 lower outlier: 0.08 years (1 month)
3 lower outliers: 47, 47 & 50 years
B
MSI-CE-MS Analysis
Mean ± 2 SD
Mean ± 3 SD
1 upper outlier: 1 year (12 months)
3 upper outliers: 42 years
1 upper outlier: 75 years
n: 183
n: 57
n: 66
n: 60
1 lower outlier: 0.08 years (1 month)
3 lower outliers: 47, 47 & 50 years
C
16S rRNA +Shotgun Metagenomic Sequencing
Mean ± 2 SD
Mean ± 3 SD
1 upper outlier: 1 year (12 months)
2 upper outliers: 42 years
n: 128
n: 40
n: 45
n: 43
1 lower outlier: 0.17 years (2 months)
2 lower outliers: 47 & 51 years

Figure S2: Graphical representation of age distributions and age outliers in all techniques. A: Age outliers in GC-QTOF-MS dataset. **B:** Age outliers in MSI-CE-MS dataset. **C:** Age outliers in Genomics (16S rRNA sequencing + shotgun metagenomics) dataset. Numbers of samples used A and B (n) are shown in each figure. Figures represent all samples in each age group, with the central line representing mean age of each group, error bars showing ± standard deviation (SD), lighter pink shaded area ± 2SD and darker pink ± 3SD. Samples lying outside the Mean ± 2SD area were considered age outliers and were excluded from further analyses. Source data are provided as a Source Data file. QC: quality control sample.

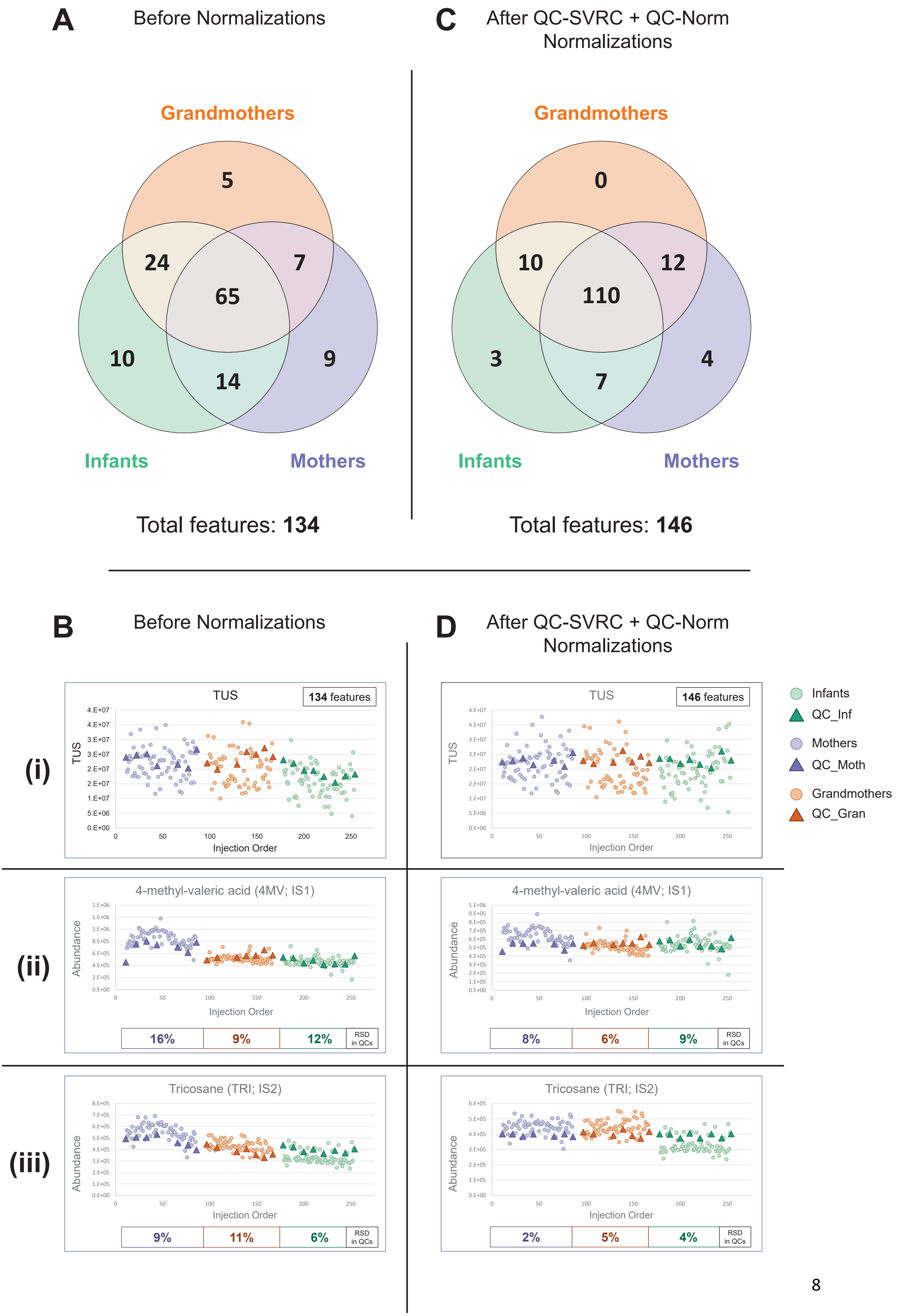
A
Before Normalizations
Grandmothers
Infants
Mothers
5
24
7
65
10
14
9
Total features: 134
C
After QC-SVRC + QC-Norm Normalizations
Grandmothers
Infants
Mothers
0
10
12
110
3
7
4
Total features: 146
B
Before Normalizations
D
After QC-SVRC + QC-Norm Normalizations
(i)
TUS
134 features
146 features
Injection Order
Infants
QC_Inf
Mothers
QC_Moth
Grandmothers
QC_Gran
(ii)
4-methyl-valeric acid (4MV; IS1)
Abundance
16%
9%
12%
RSD in QCs
8%
6%
9%
(iii)
Tricosane (TRI; IS2)
9%
11%
6%
2%
5%
4%

Figure S3: Quality Assurance and normalizations in GC-QTOF-MS analysis. A: Venn diagram of the number of features that passed the QA in each batch before normalizations, after the analysis of n = 67, n = 63, n = 65 of biologically independent samples for mothers, grandmothers, and infants, respectively. **B:** Quality parameters before normalizations, plotted by injection order: **(i)** Total Useful Signal (TUS), the sum of the abundances of all features that pass the QA in at least one batch; Internal Standards (IS): **(ii)** 4-methylvaleric acid (4MV), IS1, the IS that is added before the derivatization and measures the variability of the sample preparation as well as the analytical variability; **(iii)** tricosane (TRI), IS2, the IS that is added after derivatization and measures mostly the variability of the instrumental performance. **C:** Venn diagram of the number of features that passed the QA in each batch after the normalizations after the analysis of n = 67, n = 63, n = 65 of biologically independent samples for mothers, grandmothers, and infants, respectively. **D:** Quality parameters after the normalizations, plotted by injection order: **(i)** TUS; Internal Standards (IS): **(ii)** 4MV, IS1; **(iii)** TRI, IS2. Source data are provided as a Source Data file.

Graphs in **B** and **D** are plotted by injection order and show the three batches in the order they were measured: n = 67, n = 63, n = 65 of biologically independent samples for mothers, grandmothers, and infants, respectively. In general, the figures show that the normalization strategy was successful, as it reduced the analytical variability between the three batches – as is evidenced by the reduction in the RSD for the IS – and allowed a higher number of features to pass the QA.

RSD: relative standard deviation (also known as CV: coefficient of variance). 4MV: 4-methylvaleric acid; TRI: tricosane; QCs: quality control samples.

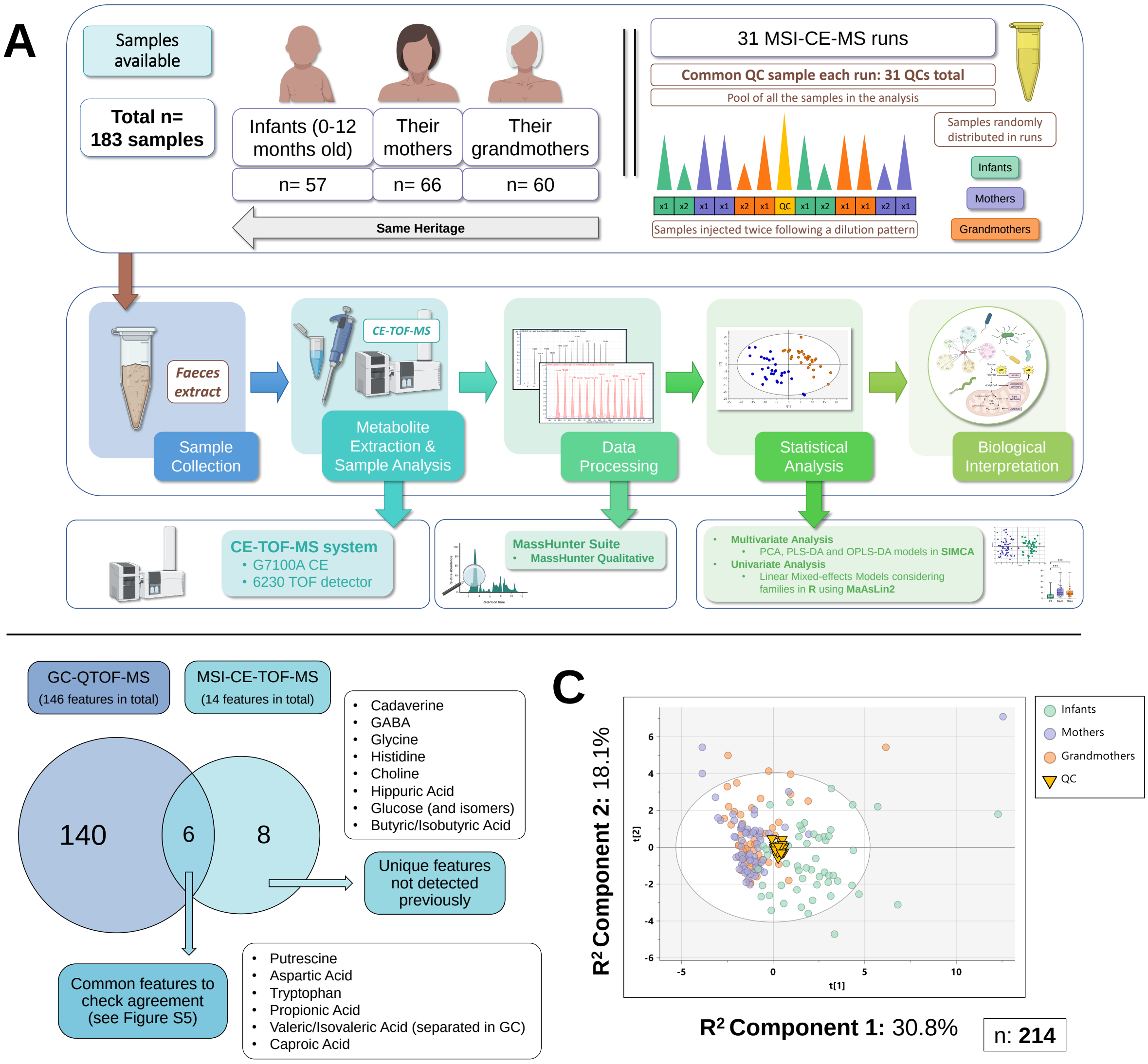

Figure S4: MSI-CE-MS workflow and multivariate models. A: Experimental design of the study, analysis, and workflow schematic. **B:** Venn diagram of the features detected in each metabolomics technique, features from GC-QTOF-MS were after the analysis of n = 63, n = 64, n = 58 of biologically independent samples for infants, mothers, and grandmothers, respectively, whereas features from MSI-CE-MS were obtained after the analysis of n = 54, n = 62, n = 55 of biologically independent samples for infants, mothers, and grandmothers, respectively. The 14 metabolites detected in a targeted search in MSI-CE-TOF-MS are shown, and further details about these metabolites are given in Supplementary Data 2. **C:** Unsupervised multivariate PCA-X model of all samples, including QCs, which are shown tightly clustered in the center. Number of samples used to build the model (n) is shown in the figure. The full explanation for sample numbers is presented in Figure S1 and S2. Source data are provided as a Source Data file. Icons were created using biorender.com.

R^2: percentage of the variability of the samples that the selected component is able to explain. Values of R^2 closer to 1 indicate higher quality of the model. All multivariate models were built with univariate scaling (UV) and without any transformation of the data.

QC: quality control sample; PCA: Principal Component Analysis; PLSDA: Partial Least Square Discriminant Analysis, OPLSDA; Orthogonal PLSDA.

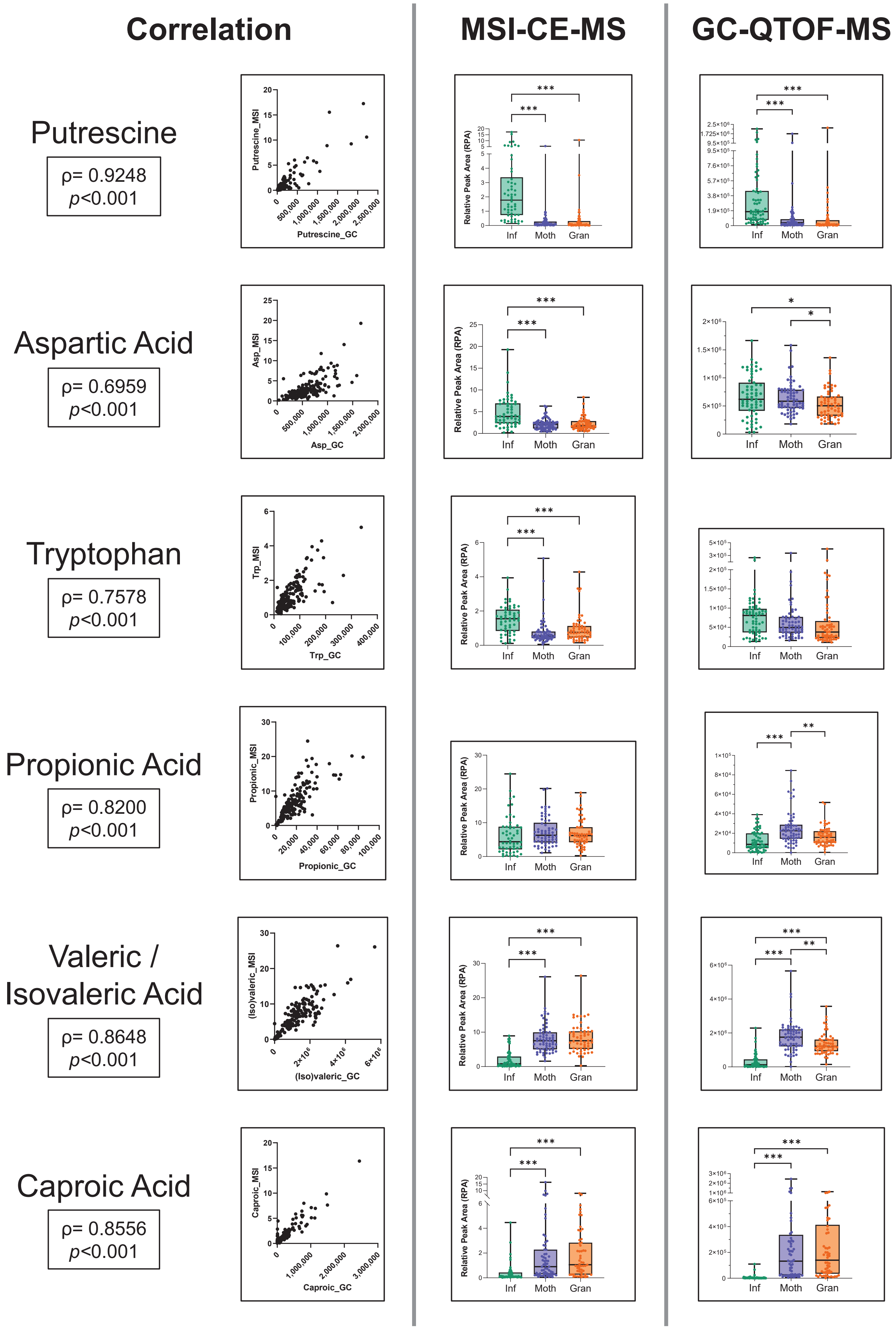
Correlation
MSI-CE-MS
GC-QTOF-MS
Putrescine
ρ= 0.9248
p<0.001
Putrescine_MSI
Putrescine_GC
Aspartic Acid
ρ= 0.6959
p<0.001
Asp_MSI
Asp_GC
Tryptophan
ρ= 0.7578
p<0.001
Trp_MSI
Trp_GC
Propionic Acid
ρ= 0.8200
p<0.001
Propionic_MSI
Propionic_GC
Valeric / Isovaleric Acid
ρ= 0.8648
p<0.001
(Iso)valeric_MSI
(Iso)valeric_GC
Caproic Acid
ρ= 0.8556
p<0.001
Caproic_MSI
Caproic_GC
Relative Peak Area (RPA)
Inf
Moth
Gran

Figure S5: Correlations between the metabolites that were measured in both techniques (MSI-CE-MS and GC-QTOF-MS) using spearman's rank correlation coefficient. Correlations were calculated with the n = 169 of the common biologically independent samples between analytical techniques. ρ: spearman rho correlation value. *p*: *p*-value of the correlation. MSI-CE-MS plots with the n = 171 of the total biologically independent samples including the Infants, Mothers and Grandmothers groups. GC-QTOF-MS plots with the n= 185 of the total biologically independent samples including the Infants, Mothers and Grandmothers groups. Statistical test was linear mixed-effects model with the correction for multiple test comparisons, FDR p-value < 0.05. *: $p<0.05$; **: $p<0.01$; ***: $p<0.001$, exact p-values and FDR p-values are provided in Supplementary Data 1 and 2. Source data are provided as a Source Data file.

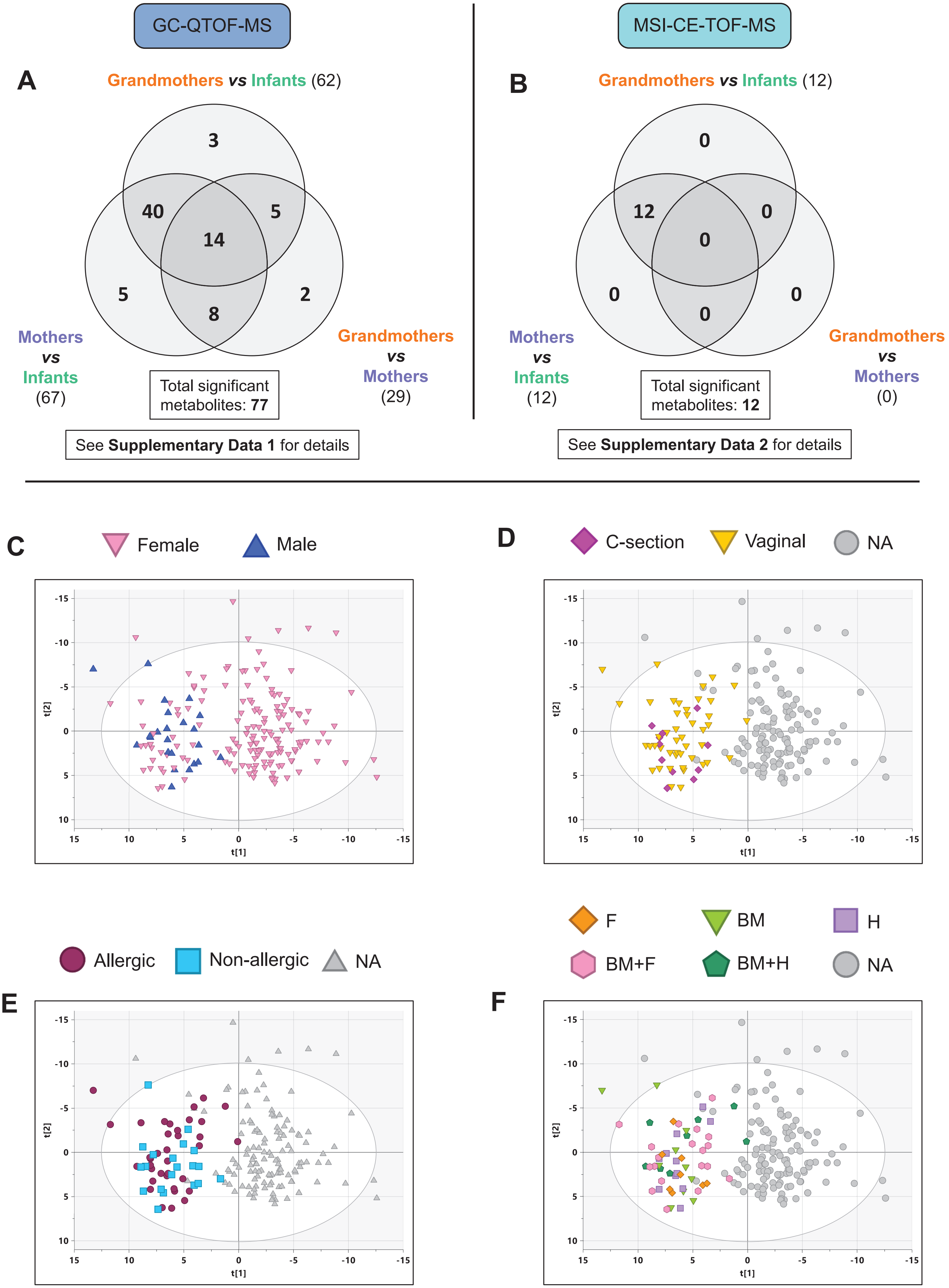
GC-QTOF-MS
A
Grandmothers vs Infants (62)
3
40
5
14
5
2
8
Mothers vs Infants (67)
Grandmothers vs Mothers (29)
Total significant metabolites: 77
See Supplementary Data 1 for details
MSI-CE-TOF-MS
B
Grandmothers vs Infants (12)
0
12
0
0
0
0
0
Mothers vs Infants (12)
Grandmothers vs Mothers (0)
Total significant metabolites: 12
See Supplementary Data 2 for details
C
Female
Male
D
C-section
Vaginal
NA
E
Allergic
Non-allergic
NA
F
F
BM
H
BM+F
BM+H
NA
t[1]
t[2]

Figure S6: Supplementary statistics figures for metabolomics. A-B: Venn diagrams of the significant metabolites according to univariate statistics for the three comparisons for (**A**) GC-QTOF-MS data, and (**B**) MSI-CE-TOF-MS data. The statistical test was linear mixed-effects model with the correction for multiple test comparisons, FDR p-value < 0.05. Exact p-values, FDR p-values, and detailed information are provided in Supplementary Data 1 and 2. **C-F:** PCA models built with the combined metabolomics data, such as the one shown in Figure 2A. For these models, the number of samples are n = 33, n = 43, n = 37 of biologically independent samples for infants, mothers, and grandmothers, respectively. In these models, the samples are colored according to different covariables: sex (**C**), mode of birth (**D**), cow's milk allergy (**E**) and type of feeding of the infant (**F**). The feeding types are named as follows: formula (F), breast milk (BM), hydrolyzed formula (H), combination of BM and F (BM+F) and combination of BM and H (BM+H). Source data are provided as a Source Data file.

The samples do not cluster according to these covariables, which proves that they have no impact on the differences according to the main variable (the age group).

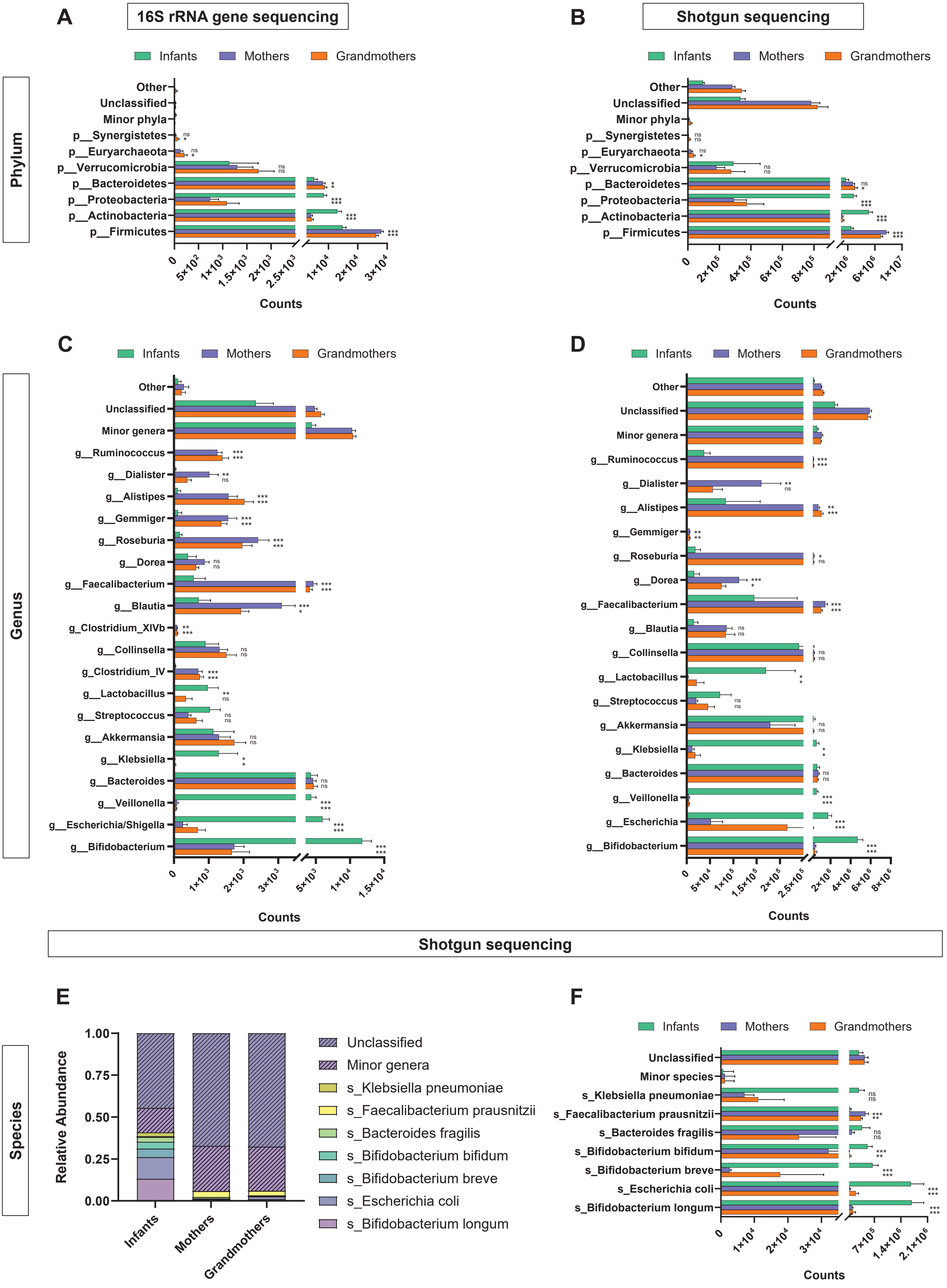
A
16S rRNA gene sequencing
B
Shotgun sequencing
Infants
Mothers
Grandmothers
Phylum
Other
Unclassified
Minor phyla
p__Synergistetes
p__Euryarchaeota
p__Verrucomicrobia
p__Bacteroidetes
p__Proteobacteria
p__Actinobacteria
p__Firmicutes
Counts
C
D
Genus
Other
Unclassified
Minor genera
g__Ruminococcus
g__Dialister
g__Alistipes
g__Gemmiger
g__Roseburia
g__Dorea
g__Faecalibacterium
g__Blautia
g__Clostridium_XIVb
g__Collinsella
g__Clostridium_IV
g__Lactobacillus
g__Streptococcus
g__Akkermansia
g__Klebsiella
g__Bacteroides
g__Veillonella
g__Escherichia/Shigella
g__Bifidobacterium
g__Escherichia
Counts
Shotgun sequencing
E
F
Species
Relative Abundance
Infants
Mothers
Grandmothers
Unclassified
Minor genera
s_Klebsiella pneumoniae
s_Faecalibacterium prausnitzii
s_Bacteroides fragilis
s_Bifidobacterium bifidum
s_Bifidobacterium breve
s_Escherichia coli
s_Bifidobacterium longum
Minor species
Counts

Figure S7: Taxonomic composition of faecal samples using 16S rRNA and shotgun gene sequencing. A-B: Relative abundance of the main **phyla** for each age group using 16S rRNA gene and shotgun sequencing, respectively. The number of samples per group are n = 38, n = 43, n = 40 of biologically independent samples for Infants, Mothers and Grandmothers groups, respectively. Data are represented as bars of the Mean + Standard Error of the Mean (SEM). Statistical test was linear mixed-effects model with the correction for multiple test comparisons, FDR p-value < 0.05. *: p<0.05; **: p<0.01; ***: p<0.001, exact p-values and FDR p-values are provided in Supplementary Data 4. **C-D:** Relative abundance of the main **genera** for each age group using 16S rRNA gene and shotgun sequencing, respectively. The number of samples per group are n = 38, n = 43, n = 40 of biologically independent samples for Infants, Mothers and Grandmothers groups, respectively. Data are represented as bars of the Mean + Standard Error of the Mean (SEM). Statistical test was linear mixed-effects model with the correction for multiple test comparisons, FDR p-value < 0.05. *: p<0.05; **: p<0.01; ***: p<0.001, exact p-values and FDR p-values are provided in Supplementary Data 5. **E:** Main **species** for each age group using shotgun sequencing. Data are represented using stacked bars. Detailed information can be found in Supplementary Data 6. **F:** Relative abundance of the main **species** for each age group using 16S rRNA gene and shotgun sequencing, respectively. The number of samples per group are n = 38, n = 43, n = 40 of biologically independent samples for Infants, Mothers and Grandmothers groups, respectively. Data are represented as bars of the Mean + Standard Error of the Mean (SEM). Statistical test was linear mixed-effects model with the correction for multiple test comparisons, FDR p-value < 0.05. *: p<0.05; **: p<0.01; ***: p<0.001, exact p-values and FDR p-values are provided in Supplementary Data 6. Source data are provided as a Source Data file.

All comparisons in sections A, B, C, D, and F, are from the adults compared to the Infants, since no significant results were obtained between Mothers and Grandmothers.

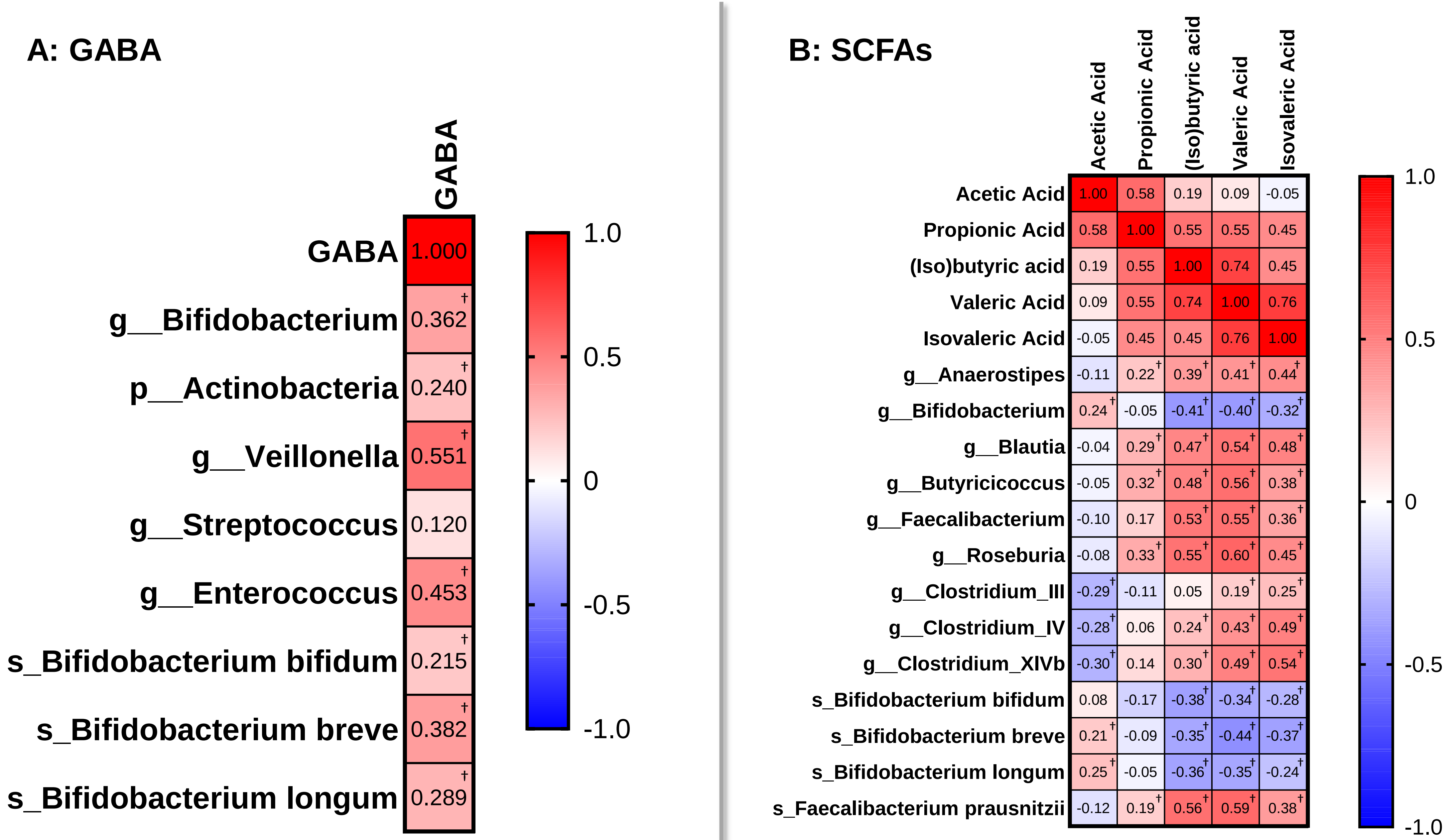
A: GABA
GABA
GABA 1.000
g__Bifidobacterium 0.362†
p__Actinobacteria 0.240†
g__Veillonella 0.551†
g__Streptococcus 0.120
g__Enterococcus 0.453†
s_Bifidobacterium bifidum 0.215†
s_Bifidobacterium breve 0.382†
s_Bifidobacterium longum 0.289†
1.0
0.5
0
-0.5
-1.0
B: SCFAs
Acetic Acid
Propionic Acid
(Iso)butyric acid
Valeric Acid
Isovaleric Acid
Acetic Acid 1.00 0.58 0.19 0.09 -0.05
Propionic Acid 0.58 1.00 0.55 0.55 0.45
(Iso)butyric acid 0.19 0.55 1.00 0.74 0.45
Valeric Acid 0.09 0.55 0.74 1.00 0.76
Isovaleric Acid -0.05 0.45 0.45 0.76 1.00
g__Anaerostipes -0.11 0.22† 0.39† 0.41† 0.44†
g__Bifidobacterium 0.24† -0.05 -0.41† -0.40† -0.32†
g__Blautia -0.04 0.29† 0.47† 0.54† 0.48†
g__Butyricicoccus -0.05 0.32† 0.48† 0.56† 0.38†
g__Faecalibacterium -0.10 0.17 0.53† 0.55† 0.36†
g__Roseburia -0.08 0.33† 0.55† 0.60† 0.45†
g__Clostridium_III -0.29† -0.11 0.05 0.19† 0.25†
g__Clostridium_IV -0.28† 0.06 0.24† 0.43† 0.49†
g__Clostridium_XIVb -0.30† 0.14 0.30† 0.49† 0.54†
s_Bifidobacterium bifidum 0.08 -0.17 -0.38† -0.34† -0.28†
s_Bifidobacterium breve 0.21† -0.09 -0.35† -0.44† -0.37†
s_Bifidobacterium longum 0.25† -0.05 -0.36† -0.35† -0.24†
s_Faecalibacterium prausnitzii -0.12 0.19† 0.56† 0.59† 0.38†
1.0
0.5
0
-0.5
-1.0

Figure S8: Correlations between metabolites and bacterial taxa that are known producers of these metabolites using spearman's rank correlation coefficient. A: Correlation between gamma-aminobutyric acid (GABA) and bacterial taxa (phyla, genera and species). The number of samples are n = 33, n = 43, n = 37 of biologically independent samples for infants, mothers, and grandmothers, respectively. Statical test was spearman's rank correlation coefficient, significant correlations (*p*-values < 0.05) are indicated with the symbol †. **B:** Correlation between short-chain fatty acids (SCFAs) and bacterial taxa (genera and species). The number of samples are n = 33, n = 43, n = 37 of biologically independent samples for infants, mothers, and grandmothers, respectively. Statical test was spearman's rank correlation coefficient, significant correlations (*p*-values < 0.05) are indicated with the symbol †. For both, A and B, the correlations are shown as heatmaps, where the Spearman rho (ρ) correlation parameter for each comparison is shown with a color code. Red boxes indicate a positive correlation and blue boxes a negative one, exact p-values are given in Table S3 for GABA and Table S4 for SCFAs. Source data are provided as a Source Data file.

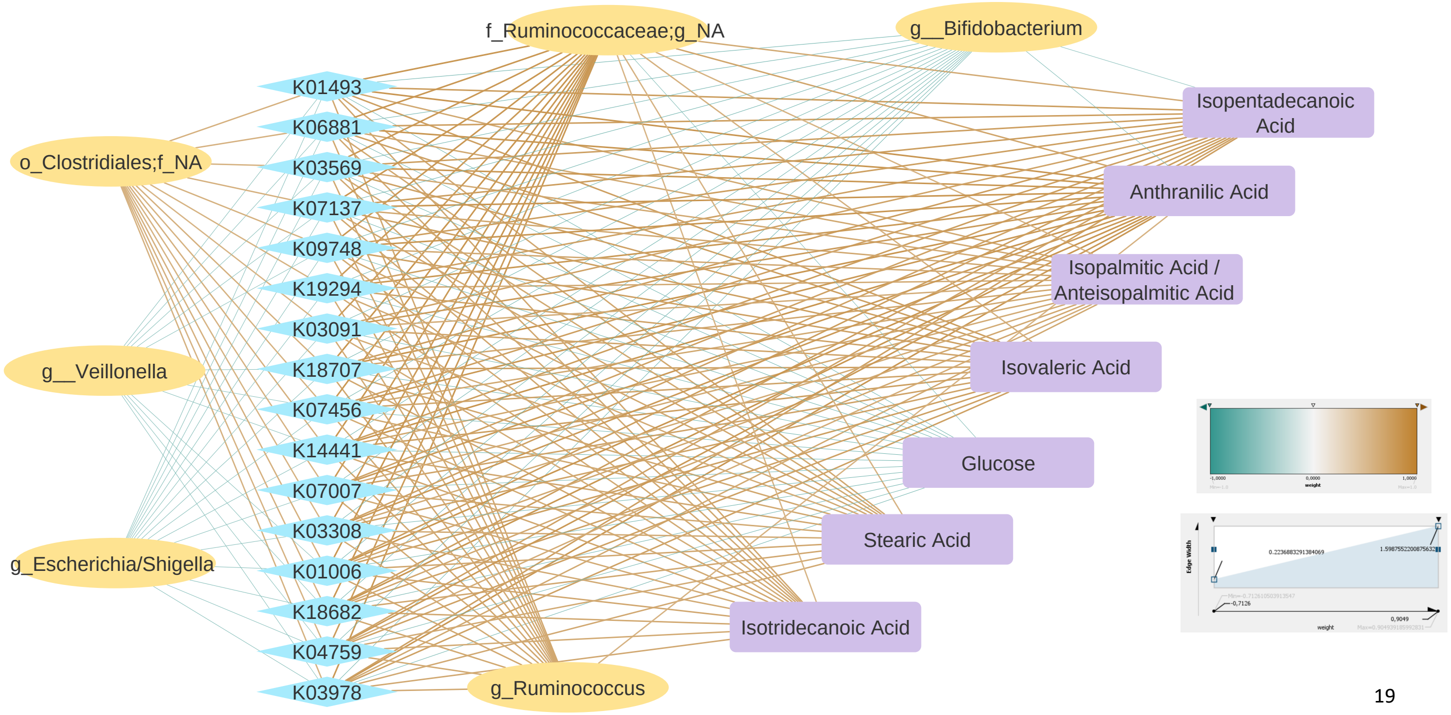
f_Ruminococcaceae;g_NA
g__Bifidobacterium
o_Clostridiales;f_NA
g__Veillonella
g_Escherichia/Shigella
g_Ruminococcus
K01493
K06881
K03569
K07137
K09748
K19294
K03091
K18707
K07456
K14441
K07007
K03308
K01006
K18682
K04759
K03978
Isopentadecanoic Acid
Anthranilic Acid
Isopalmitic Acid / Anteisopalmitic Acid
Isovaleric Acid
Glucose
Stearic Acid
Isotridecanoic Acid

Figure S9: Cytoscape representation of the links between ASVs, KOs, and metabolites obtained from mixOmics and DIABLO. ASVs are represented by the yellow ovals, KOs by the blue diamonds, and metabolites by the purple rectangles. KOs names are described in Figure 9. The blue-green edges indicate a negative correlation, while the brown edges represent a positive correlation. The width of the edge increases with the correlation weight.

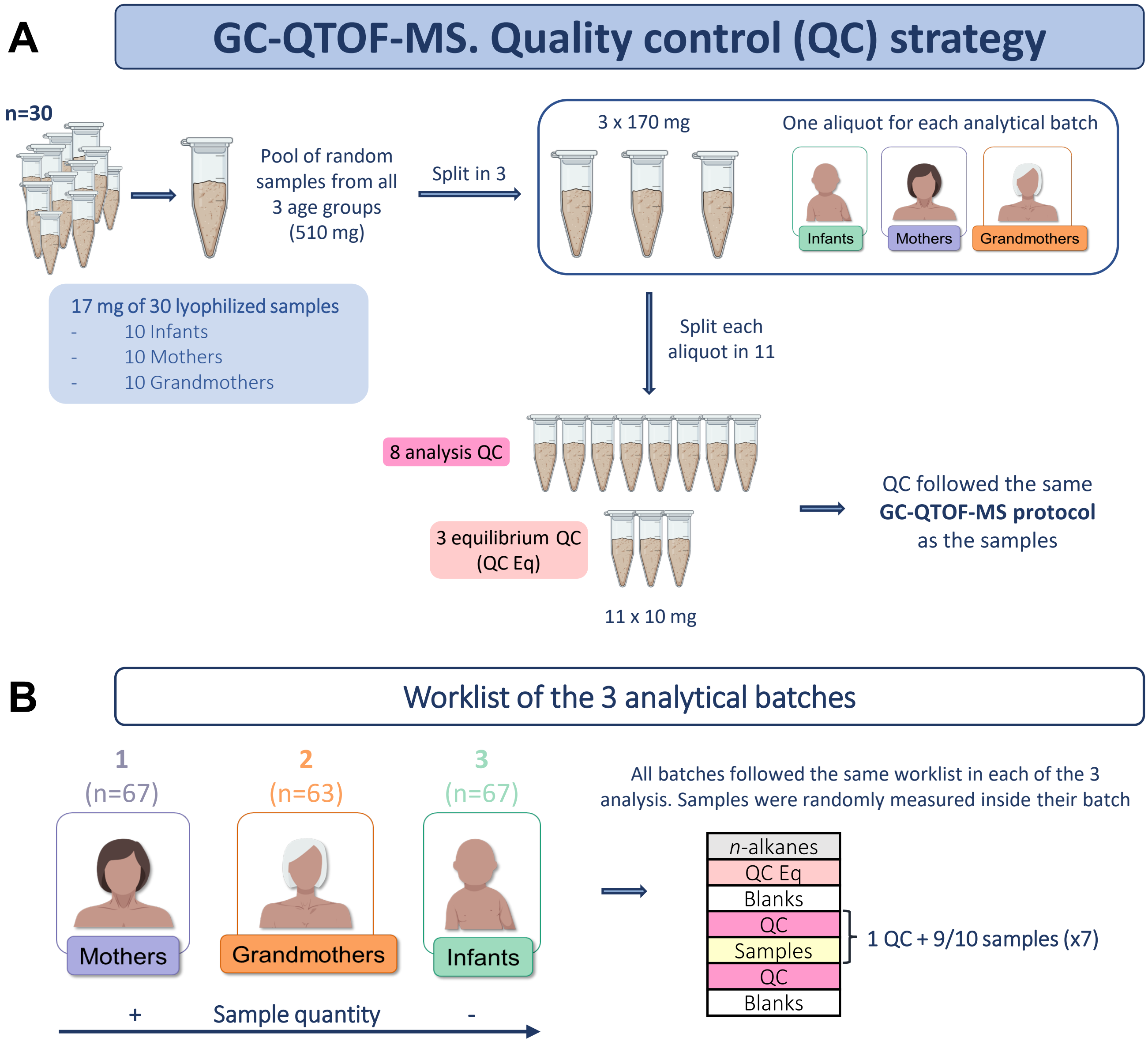

Figure S10: Experimental design of the QCs preparation and worklists for GC-QTOF-MS equipment used for large-scale sample analysis. A: Scheme of the QC strategy used in the analysis to ensure the subsequent joining of the batches through a normalization approach. **B:** Outline of the three analytical batches (Infants, Mothers and Grandmothers) and their corresponding worklists carried out on the GC-QTOF-MS equipment. QCs: quality control samples. Icons were created using biorender.com.

Table S1: Main characteristics for each age group, with their descriptive statistics.

		Infants		Mothers		Grandmothers	
		N	%	N	%	N	%
**N**		69		67		64	
**Age (mean years ± SD)**		0.42 ± 0.14		34.31 ± 3.8		63.09 ± 6.09	
**Age (mean months ± SD)**		5.03 ± 1.71		-		-	
**Age range (years)**		0.08-1		27-42		47-76	
**Age range (months)**		1-12		-		-	
**Sex**							
	Female	38	55%	67	100%	64	100%
	Male	31	45%	-	-	-	-
**Mode of birth**							
	C-section	16	23%	-		-	
	Vaginal	53	77%	-		-	
**Antibiotics during delivery**							
	No	55	80%	-		-	
	Yes	10	14%	-		-	
	NA	4	6%	-		-	
**Cow's milk allergy (Infants)**							
	Allergic	46	67%	-		-	
	Non-allergic	23	33%	-		-	
**Infant feeding**							
	Breastmilk	12	17%	-		-	
	Formula	8	12%	-		-	
	Hydrolysed formula	12	17%	-		-	
	Breastmilk + Formula	23	33%	-		-	
	Breastmilk + Hydrolysed formula	14	20%	-		-	
**Smoking habits**							
	Never	-		48	72%	36	56%
	Ever	-		19	28%	28	44%

Table S2: Metabolic Pathway Analysis results using MetaboAnalyst v6.0.

Pathway name	Match Status	p	-log(p)	Holm p	FDR	Impact
**Biosynthesis of unsaturated fatty acids**	8/36	2.24E-06	5.650	1.79E-04	1.79E-04	0.000
**Butanoate metabolism**	5/15	2.61E-05	4.583	2.06E-03	1.04E-03	0.143
Glutathione metabolism	6/28	6.08E-05	4.216	4.74E-03	1.22E-03	0.126
**Alanine, aspartate and glutamate metabolism**	6/28	6.08E-05	4.216	4.74E-03	1.22E-03	0.594
Glyoxylate and dicarboxylate metabolism	5/32	1.25E-03	2.903	9.50E-02	1.93E-02	0.138
Glycine, serine and threonine metabolism	5/33	1.44E-03	2.840	1.08E-01	1.93E-02	0.355
**Propanoate metabolism**	4/22	2.26E-03	2.646	1.67E-01	2.58E-02	0.041
**Citrate cycle (TCA cycle)**	3/20	1.47E-02	1.834	1.00E+00	1.00E-01	0.167
**Tryptophan metabolism**	2/41	2.99E-01	0.524	1.00E+00	8.25E-01	0.145

Labels of the colors: In blue: $p < 0.001$, in light green: $0.001 < p < 0.01$, in green: $0.01 < p < 0.05$, in red: $p > 0.05$. Pathways in bold font are those selected for discussion in the manuscript. Analysis was carried out using Metaboanalyst v6.0

Table S3: Correlations between gamma-aminobutyric acid (GABA) and bacterial phyla, genera, and species.

	GABA vs. g__Bifidobacterium	GABA vs. p__Actinobacteria	GABA vs. g__Veillonella	GABA vs. g__Streptococcus	GABA vs. g__Enterococcus	GABA vs. s_Bifidobacterium bifidum	GABA vs. s_Bifidobacterium breve	GABA vs. s_Bifidobacterium longum
Spearman rho (ρ)	0.3624	0.2404	0.5511	0.1202	0.4527	0.2154	0.3819	0.2889
95% confidence interval	0.1770 to 0.5230	0.04439 to 0.4186	0.3962 to 0.6755	-0.07985 to 0.3109	0.2797 to 0.5973	0.01807 to 0.3966	0.1988 to 0.5392	0.09623 to 0.4606
P (two-tailed)	0.0002	0.0140	0.0000	0.2243	0.0000	0.0281	0.0001	0.0029
P value summary	***	*	***	ns	***	*	***	**
Significant? (alpha = 0.05)	Yes	Yes	Yes	No	Yes	Yes	Yes	Yes

Spearman rho (ρ) color scale: ρ= positive values are displayed in red while negative values would be in blue, ρ=0 would be in white. Significance color scale cell, for p values < 0.05, cells are in green and p values > 0.05 are in red.

Table S4: Correlations between short-chain fatty acids (SCFA) and bacterial genera and species.

			Acetic Acid		Propionic Acid		Butyric/Isobutyric Acid		Valeric Acid		Isovaleric Acid	
		Producer of	ρ	*p*-value	ρ	*p*-value	ρ	*p*-value	ρ	*p*-value	ρ	*p*-value
SCFAs	Acetic Acid	-	1.0000		0.5799	1.69E-11	0.1921	4.15E-02	0.0890	3.49E-01	-0.0466	6.24E-01
	Propionic Acid	-	0.5799	1.69E-11	1.0000		0.5515	2.44E-10	0.5457	4.09E-10	0.4516	5.15E-07
	Butyric/Isobutyric Acid	-	0.1921	4.15E-02	0.5515	2.44E-10	1.0000		0.7358	1.64E-20	0.4505	5.52E-07
	Valeric Acid	-	0.0890	3.49E-01	0.5457	4.09E-10	0.7358	1.64E-20	1.0000		0.7590	2.05E-22
	Isovaleric Acid	-	-0.0466	6.24E-01	0.4516	5.15E-07	0.4505	5.52E-07	0.7590	2.05E-22	1.0000	
Genera	g__Anaerostipes	Butyrate	-0.1136	2.31E-01	0.2175	2.07E-02	0.3872	2.27E-05	0.4148	4.94E-06	0.4445	8.13E-07
	g__Bifidobacterium	Acetate	0.2434	9.37E-03	-0.0521	5.84E-01	-0.4067	7.80E-06	-0.3967	1.36E-05	-0.3248	4.48E-04
	g__Blautia	Propionate	-0.0424	6.55E-01	0.2893	1.88E-03	0.4717	1.34E-07	0.5392	7.18E-10	0.4824	6.30E-08
	g__Butyricicoccus	Butyrate	-0.0508	5.93E-01	0.3169	6.28E-04	0.4831	5.97E-08	0.5608	1.04E-10	0.3802	3.27E-05
	g__Faecalibacterium	Butyrate	-0.0998	2.93E-01	0.1734	6.62E-02	0.5262	2.16E-09	0.5547	1.83E-10	0.3555	1.12E-04
	g__Roseburia	Propionate and butyrate	-0.0846	3.73E-01	0.3287	3.78E-04	0.5478	3.40E-10	0.6021	1.73E-12	0.4546	4.25E-07
	g__Clostridium_III	Valerate	-0.2895	1.87E-03	-0.1135	2.31E-01	0.0531	5.76E-01	0.1936	3.99E-02	0.2528	6.91E-03
	g__Clostridium_IV	Valerate	-0.2777	2.90E-03	0.0649	4.95E-01	0.2440	9.21E-03	0.4254	2.64E-06	0.4940	2.70E-08
	g__Clostridium_XlVb	Valerate	-0.2987	1.31E-03	0.1391	1.42E-01	0.3015	1.18E-03	0.4885	4.05E-08	0.5404	6.48E-10
Species	s_Bifidobacterium bifidum	Acetate	0.0767	4.20E-01	-0.1726	6.75E-02	-0.3760	4.05E-05	-0.3411	2.18E-04	-0.2836	2.33E-03
	s_Bifidobacterium breve	Acetate	0.2077	2.73E-02	-0.0885	3.51E-01	-0.3484	1.56E-04	-0.4405	1.05E-06	-0.3715	5.11E-05
	s_Bifidobacterium longum	Acetate	0.2467	8.44E-03	-0.0451	6.35E-01	-0.3625	7.96E-05	-0.3469	1.67E-04	-0.2395	1.06E-02
	s_Faecalibacterium prausnitzii	Butyrate	-0.1199	2.06E-01	0.1857	4.90E-02	0.5600	1.13E-10	0.5859	9.32E-12	0.3849	2.56E-05

Spearman rho (ρ) color scale: ρ= positive values are displayed in red while negative values would be in blue, ρ=0 would be in white. Significance color scale cell, for p values < 0.05, cells are in green and p values > 0.05 are in red.

Table S5: Correlations between the matched bacterial genera between 16S rRNA gene sequencing and shotgun sequencing.

Genera	p.value_Pearson	ρ Pearson
g_Caryophanon	0.00E+00	**1.000**
g_Hafnia	3.88E-94	**0.989**
g_Megasphaera	2.16E-85	**0.984**
g_Acidaminococcus	8.86E-84	**0.983**
g_Slackia	8.34E-77	**0.977**
g_Klebsiella	7.63E-71	**0.971**
g_Parasutterella	1.29E-69	**0.969**
g_Bilophila	6.51E-68	**0.967**
g_Collinsella	1.10E-67	**0.967**
g_Catenibacterium	1.34E-66	**0.965**
g_Citrobacter	3.43E-66	**0.965**
g_Dialister	3.87E-64	**0.962**
g_Parabacteroides	2.32E-63	**0.960**
g_Desulfovibrio	2.12E-62	**0.959**
g_Butyricimonas	1.89E-61	**0.957**
g_Prevotella	2.95E-61	**0.957**
g_Hungatella	4.03E-61	**0.956**
g_Alistipes	2.35E-57	**0.949**
g_Streptococcus	6.25E-57	**0.948**
g_Odoribacter	1.31E-56	**0.947**
g_Senegalimassilia	9.13E-56	**0.945**
g_Enterococcus	3.94E-55	**0.944**
g_Bifidobacterium	4.79E-55	**0.943**
g_Paraprevotella	3.46E-54	**0.941**
g_Lactococcus	1.52E-52	**0.937**
g_Megamonas	1.47E-48	**0.925**
g_Eggerthella	8.24E-47	**0.919**
g_Akkermansia	1.56E-46	**0.918**
g_Faecalibacterium	5.81E-46	**0.916**
g_Flavonifractor	3.41E-44	**0.910**
g_Lactobacillus	3.75E-42	**0.901**
g_Haemophilus	4.28E-40	**0.892**
g_Methanobrevibacter	6.62E-40	**0.891**
g_Butyricicoccus	1.23E-38	**0.885**
g_Faecalitalea	6.09E-38	**0.881**
g_Veillonella	1.69E-37	**0.879**
g_Gordonibacter	2.13E-36	**0.873**
g_Ruminococcus	7.62E-33	**0.851**
g_Romboutsia	1.63E-30	**0.835**
g_Granulicatella	3.62E-30	**0.832**
g_Fusicatenibacter	2.04E-29	**0.826**
g_Phascolarctobacterium	1.59E-27	**0.810**

g_Gemmiger	9.88E-27	**0.803**
g_Holdemanella	5.53E-26	**0.796**
g_Coprobacillus	4.59E-25	**0.787**
g_Adlercreutzia	1.02E-24	**0.784**
g_Turicibacter	1.65E-24	**0.782**
g_Coprococcus	2.11E-24	**0.781**
g_Olsenella	6.06E-24	**0.776**
g_Bacteroides	8.11E-24	**0.775**
g_Butyrivibrio	2.86E-23	**0.769**
g_Blautia	5.91E-21	**0.741**
g_Enterobacter	1.12E-20	**0.738**
g_Intestinibacter	7.92E-20	**0.727**
g_Actinomyces	1.44E-18	**0.709**
g_Oscillibacter	1.18E-17	**0.696**
g_Barnesiella	2.39E-16	**0.675**
g_Roseburia	2.65E-15	**0.657**
g_Succiniclasticum	3.08E-15	**0.656**
g_Cloacibacillus	8.96E-15	**0.648**
g_Christensenella	7.00E-14	**0.631**
g_Sutterella	9.76E-13	**0.607**
g_Dorea	1.58E-11	**0.581**
g_Anaerostipes	1.81E-10	**0.555**
g_Faecalicoccus	2.80E-08	**0.493**
g_Terrisporobacter	6.89E-08	**0.481**
g_Anaerotruncus	8.53E-07	**0.444**
g_Sporobacter	1.81E-05	**0.391**
g_Anaerovorax	1.25E-04	**0.353**
g_Eisenbergiella	1.23E-03	**0.300**
g_Asaccharobacter	6.37E-03	**0.255**
g_Subdoligranulum	8.58E-03	**0.246**
g_Pseudoflavonifractor	1.04E-02	**0.240**
g_Mogibacterium	7.31E-02	0.169
g_Intestinimonas	3.68E-01	0.086
g_Alloprevotella	5.32E-01	0.059
g_Coprobacter	5.62E-01	0.055
g_Peptococcus	5.83E-01	0.052
g_Acetanaerobacterium	7.04E-01	0.036
g_Salmonella	7.24E-01	-0.034
g_Anaeroglobus	7.50E-01	-0.030
g_Enterorhabdus	8.69E-01	-0.016
g_Eubacterium	9.40E-01	-0.007
g_Hespellia	9.60E-01	0.005

Pearson correlation (ρ) color scale: ρ= positive values are displayed in red while values near to 0 or negative are in white to light blue. Significance color scale cell, for p values < 0.05, cells are in green and p values > 0.05 are in white.

Table S6: Description of the KOs from Heatmap and circosplot from Figure 9.

Order	feature	Description
1	K06881	bifunctional oligoribonuclease and PAP phosphatase NrnA [EC:3.1.3.7 3.1.13.3]
2	K01493	dCMP deaminase [EC:3.5.4.12]
3	K18682	ribonucrease Y [EC:3.1.-.-]
4	K18707	threonylcarbamoyladenosine tRNA methylthiotransferase MtaB [EC:2.8.4.5]
5	K15633	2,3-bisphosphoglycerate-independent phosphoglycerate mutase [EC:5.4.2.12]
6	K14441	ribosomal protein S12 methylthiotransferase [EC:2.8.4.4]
7	K07137	uncharacterized protein
8	K01006	pyruvate, orthophosphate dikinase [EC:2.7.9.1]
9	K22132	tRNA threonylcarbamoyladenosine dehydratase
10	K03569	rod shape-determining protein MreB and related proteins
11	K03978	GTP-binding protein
12	K09748	ribosome maturation factor RimP
13	K07456	DNA mismatch repair protein MutS2
14	K11749	regulator of sigma E protease [EC:3.4.24.-]
15	K01808	ribose 5-phosphate isomerase B [EC:5.3.1.6]
16	K19294	alginate O-acetyltransferase complex protein AlgI
17	K03499	trk system potassium uptake protein
18	K03308	neurotransmitter:Na+ symporter, NSS family
19	K07007	3-dehydro-bile acid Delta4,6-reductase [EC:1.3.1.114]
20	K07095	uncharacterized protein
21	K11175	phosphoribosylglycinamide formyltransferase 1 [EC:2.1.2.2]
22	K04759	ferrous iron transport protein B
23	K02238	competence protein ComEC
24	K07699	two-component system, response regulator, stage 0 sporulation protein A
25	K03091	RNA polymerase sporulation-specific sigma factor
