# Supplementary material for: Comparative characterization of the infant gut microbiome and their maternal lineage by a multi-omics approach
Source: Nat Commun. 2024 Apr 8;15:3004. doi: 10.1038/s41467-024-47182-y (PMC11001937; doi:10.1038/s41467-024-47182-y)
Supplement: Supplementary file 3 — Description of Additional Supplementary Files [file 41467_2024_47182_MOESM3_ESM.pdf]

Supplementary Data files:

Supplementary Data 1: Significant identified metabolites detected in the GC-QTOF-MS analysis, with their chemical information, quality parameters and statistics. The statistical test applied was linear mixed-effects model with the correction for multiple test comparisons, FDR p-value < 0.05.

Supplementary Data 2: Significant metabolites detected in the MSI-CE-TOF-MS analysis, with their chemical information, quality parameters and statistics. The statistical test applied was linear mixed-effects model with the correction for multiple test comparisons, FDR p-value < 0.05.

Supplementary Data 3: Comparisons between the bacterial phyla and genera detected by 16S rRNA gene and shotgun sequencing. Data used for the stacked bars in Figure 7A-D.

Supplementary Data 4: Significant results at phylum level for 16S rRNA gene and shotgun sequencing. The statistical test applied was linear mixed-effects model with the correction for multiple test comparisons, FDR p-value < 0.05.

Supplementary Data 5: Significant results at genus level for 16S rRNA gene and shotgun sequencing. The statistical test applied was linear mixed-effects model with the correction for multiple test comparisons, FDR p-value < 0.05.

Supplementary Data 6: Significant results at species level for shotgun sequencing. The statistical test applied was linear mixed-effects model with the correction for multiple test comparisons, FDR p-value < 0.05.

Supplementary Data 7: Significant results from the linear mixed effects model for the KEGG Orthologs (KOs).

Supplementary Data 8: Significant results from the linear mixed effects model for the pathways at BRITE level B.

Supplementary Data 9: Significant results from the linear mixed effects model for the pathways at BRITE level C.

Supplementary Data 10: Detailed information for each sample, including epidemiological variables, techniques in which it was measured and models where it is included.
