# Supplementary material for: Comparative characterization of the infant gut microbiome and their maternal lineage by a multi-omics approach
Source: Nat Commun. 2024 Apr 8;15:3004. doi: 10.1038/s41467-024-47182-y (PMC11001937; doi:10.1038/s41467-024-47182-y)
Supplement: Supplementary file 14 — Reporting Summary [file 41467_2024_47182_MOESM14_ESM.pdf]

nature portfolio

Corresponding author(s): Alma Villaseñor, PhD

Last updated by author(s): 03/13/2024

Reporting Summary

Nature Portfolio wishes to improve the reproducibility of the work that we publish. This form provides structure for consistency and transparency in reporting. For further information on Nature Portfolio policies, see our Editorial Policies and the Editorial Policy Checklist.

Statistics

For all statistical analyses, confirm that the following items are present in the figure legend, table legend, main text, or Methods section.

n/a	Confirmed	
[ ]	[x]	The exact sample size (*n*) for each experimental group/condition, given as a discrete number and unit of measurement
[x]	[ ]	A statement on whether measurements were taken from distinct samples or whether the same sample was measured repeatedly
[ ]	[x]	The statistical test(s) used AND whether they are one- or two-sided<br>*Only common tests should be described solely by name; describe more complex techniques in the Methods section.*
[x]	[ ]	A description of all covariates tested
[ ]	[x]	A description of any assumptions or corrections, such as tests of normality and adjustment for multiple comparisons
[ ]	[x]	A full description of the statistical parameters including central tendency (e.g. means) or other basic estimates (e.g. regression coefficient) AND variation (e.g. standard deviation) or associated estimates of uncertainty (e.g. confidence intervals)
[ ]	[x]	For null hypothesis testing, the test statistic (e.g. *F*, *t*, *r*) with confidence intervals, effect sizes, degrees of freedom and *P* value noted<br>*Give P values as exact values whenever suitable.*
[x]	[ ]	For Bayesian analysis, information on the choice of priors and Markov chain Monte Carlo settings
[x]	[ ]	For hierarchical and complex designs, identification of the appropriate level for tests and full reporting of outcomes
[ ]	[x]	Estimates of effect sizes (e.g. Cohen's *d*, Pearson's *r*), indicating how they were calculated

Our web collection on statistics for biologists contains articles on many of the points above.

Software and code

Policy information about availability of computer code

Data collection	Metabolomics: data collection was performed with the Agilent vendor acquisition software specific to each equipment, detailed in Materials and methods. Metagenomics: The MiSeq platform was used for 16S rRNA gene sequencing while the NextSeq platform was utilized for shotgun sequencing, both from Illumina (USA).
Data analysis	Metabolomics raw data were analyzed with the Agilent MassHunter Suite (B.10.0). Different programs were used depending on the process, as detailed in Materials and Methods: MassHunter Quantitative (B.10.0, Agilent), MassHunter Qualitative (B.08.00, Agilent), and MassHunter Unknowns Analysis (B.10.0, Agilent), for missing values KNN algorithm used MATLAB (v. 2022b). Filtering, normalization, and quality assurance were performed in Microsoft Excel for Microsoft 365 MSO (v. 2401). Statistical analyses were performed in Graph Pad Prism (v.9.5.0), SIMCA (v.16.0.1, Sartorius Stedim Data Analytics AB), R (v. 4.2.2), and MetaboAnalyst (v.6.0). Details are available in the manuscript for each specific process. 16S rRNA sequences were denoised and processed with DADA2 vl.11. Statistical analyses were performed in R (v. 4.2.2, vegan v2.6-4 & fdr.R packages), Graph Pad Prism (v.9.5.0), and LEfSe tool from Hutlab's Galaxy platform. Shotgun metagenomics sequencing data treatment was analysed with Fastp application (v. 0.20.1), and SqueezeMeta pipeline with coassembly mode (v. 1.3.1). Statistical analyses were performed in R (v. 4.2.2, MaAslin2 package). Omics datasets integration was performed with the mixOmics script using DIABLO framework using R (v. 4.2.2). The Microsoft Office suite was used extensively for writing the manuscript and creating figures and tables. Biorender and Inkscape were also used for graphics processing. Molecular structures were drawn in Chem Draw.

For manuscripts utilizing custom algorithms or software that are central to the research but not yet described in published literature, software must be made available to editors and reviewers. We strongly encourage code deposition in a community repository (e.g. GitHub). See the Nature Portfolio guidelines for submitting code & software for further information.

Data

Policy information about availability of data

All manuscripts must include a data availability statement. This statement should provide the following information, where applicable:

- Accession codes, unique identifiers, or web links for publicly available datasets
- A description of any restrictions on data availability
- For clinical datasets or third party data, please ensure that the statement adheres to our policy

Data from the patients is given in Supplementary Data 10.
The 16S rRNA and shotgun metagenomics sequencing files and metadata data generated in this study have been deposited in the Sequence Read Archive (SRA) at NIH database under accession code PRJNA991269 [https://www.ncbi.nlm.nih.gov/bioproject/PRJNA991269/]. The metabolomics raw data generated in this study have been deposited to the EMBL-EBI MetaboLights database under accession code MTBLS7670 [https://www.ebi.ac.uk/metabolights/MTBLS7670].

Research involving human participants, their data, or biological material

Policy information about studies with human participants or human data. See also policy information about sex, gender (identity/presentation), and sexual orientation and race, ethnicity and racism.

Reporting on sex and gender	Due to the design of the study itself, which is interested in maternal transference of microbiota, mothers and grandmothers were selected. Thus, all the adult population was of the female sex. The infants were not selected based on any sex bias, and their proportions were similar (55% females, 45% males) as shown in Table SI. We show in Figure S6 that sex was not a relevant covariable between the infants, as it did not create any clusters between them.
Reporting on race, ethnicity, or other socially relevant groupings	Race/ethnicity was not considered an important variable in this study, and was neither an inclusion nor exclusion criteria.
Population characteristics	The participant variables recorded for the data analysis were age, gender, mode of birth, antibiotics use at birt,I h cow's milk allergy and feeding regime for Infants, and age and smoking habits of Mothers and Grandmothers. From these, the only independent-relevant variable for the population was age, which was the variable, aim of the study. Full population characteristics are provided in the manuscript in materials and methods, and in Supplementary Data 10.
Recruitment	ubjects were enrolled in an intergenerational and observational study from 2017 until 2019 based on their arrival at the participating hospitals. While this method aimed to include a representative sample, there may be self-selection bias present. However, to avoid any bias or any impact in the results, we included possible covariates (variables) from all the population groups to control it. This was demonstrated after their analysis within the groups, and finding no differences. Faeces were collected and stored with a sample collection kit developed by our group. The inclusion criteria required that Infants were younger than 12 months of age and had not transitioned to a solid diet, and that Mothers and Grandmothers were not following any specific diet. Exclusion criteria for all age groups were antibiotics intake in the 3 months prior to study recruitment and any other concomitant severe disease.
Ethics oversight	The study was approved by the Regional Ethics Committee for Clinical Research of Hospital Universitario lnfantil Niño Jesus in Madrid according to the ethical guidelines outlined in the Declaration of Helsinki and its amendments.

Note that full information on the approval of the study protocol must also be provided in the manuscript.

Field-specific reporting

Please select the one below that is the best fit for your research. If you are not sure, read the appropriate sections before making your selection.

[x] Life sciences   [ ] Behavioural & social sciences   [ ] Ecological, evolutionary & environmental sciences

For a reference copy of the document with all sections, see nature.com/documents/nr-reporting-summary-flat.pdf

Life sciences study design

All studies must disclose on these points even when the disclosure is negative.

Sample size	Sample size was not predetermined based on power analysis. Previous studies on short chain fatty acids (SCFAs) and other metabolites in humans showed interesting biological results between infants and adults (DOI: 10.3389/fmicb.2020.00973 and 10.1021/acs.jproteome.1c00220). These studies reported a number of samples of approximately 200. So, following a similar approach, the sample size was 200 and was chosen according to the recruitment potential for the study location. In addition, laboratory logistics and capability for this study were also taken into consideration.
Data exclusions	In some steps of the analyses, due to insufficient quantity or equipment malfunction, some samples were lost. For this reason, a different number of samples was available for each technique. All these issues and processes are detailed in the manuscript and in Materials and Methods, and the specific samples used for each step are shown in Figure Sl and Supplementary Data 10. Samples from each age group that presented an age higher than [Mean+2SD] or lower than [Mean-2SD] were deemed age outliers and thus excluded from further analysis (Figure S2). Other samples were also excluded from further statistical analysis if they were clear outliers in the multivariate models.

Replication	The samples that had enough quantity were measured in several techniques as part of our multi-omics strategy. These analyses were performed independently in each omics platform, i.e. GC-QTOF-MS, MSI-CE-MS, 16S rRNA and shotgun gene sequencing. In metabolomics, 6 metabolites that had been detected in GC-QTOF-MS were also obtained in MSI-CE-TOF-MS and their relative abundances showed positive significant correlation demonstrating successful replication analysis (Figure S5). For the metabolomics platforms, reproducibility is ensured by injecting identical QC samples into both techniques, and this process was successfully carried out for each of the three batches (infants, mothers, and grandmothers) as shown in Figure S9. In the case of 16S rRNA and shotgun gene sequencing, DNA quality and amount are evaluated before analysis.
Randomization	For metabolomics, allocation into groups was not random because it was inseparable from the characteristics of each group (Infant/Mother/Grandmother). The order of samples was randomized in each group to avoid any analytical bias. This was confirmed a QC sample from all groups was consistently measured throughout the analysis for 16S rRNA and shotgun gene sequence analysis, all samples were blinded and randomized.
Blinding	We did not consider blinding relevant in this study, as the study was observational and the individuals did not received any treatment. The mother collected the samples for her and their infant, as well as for the grandmother. The medical team needed to revised the labels of the collected sample. The analyst for metabolomics, needed to know which group each sample belonged to because randomization was conducted within each group. However, this did not affect the study because the samples were randomized, and a QC sample from all groups was consistently measured throughout the analysis. On the other hand, for 16S rRNA and shotgun gene sequence analysis, all samples were blinded and randomized, until statistical analysis.

Reporting for specific materials, systems and methods

We require information from authors about some types of materials, experimental systems and methods used in many studies. Here, indicate whether each material, system or method listed is relevant to your study. If you are not sure if a list item applies to your research, read the appropriate section before selecting a response.

Materials & experimental systems

n/a	Involved in the study	
☒	☐	Antibodies
☒	☐	Eukaryotic cell lines
☒	☐	Palaeontology and archaeology
☒	☐	Animals and other organisms
☒	☐	Clinical data
☒	☐	Dual use research of concern
☒	☐	Plants

Methods

n/a	Involved in the study	
☒	☐	ChIP-seq
☒	☐	Flow cytometry
☒	☐	MRI-based neuroimaging
